# Research reviews and prospects of gut microbiota in liver cirrhosis: a bibliometric analysis (2001–2023)

**DOI:** 10.3389/fmicb.2024.1342356

**Published:** 2024-03-14

**Authors:** Xiaofei Zhu, Ziyuan Zhou, Xiaxia Pan

**Affiliations:** ^1^Department of Infectious Diseases, Hangzhou Ninth People’s Hospital, Hangzhou, China; ^2^State Key Laboratory for Diagnosis and Treatment of Infectious Diseases, National Clinical Research Center for Infectious Diseases, Collaborative Innovation Center for Diagnosis and Treatment of Infectious Diseases, The First Affiliated Hospital, Zhejiang University School of Medicine, Hangzhou, China; ^3^Cancer Center, Department of Pulmonary and Critical Care Medicine, Zhejiang Provincial People’s Hospital (Affiliated People’s Hospital), Hangzhou Medical College, Hangzhou, China

**Keywords:** liver cirrhosis, gut microbiota, bibliometrics, VOSviewers, CiteSpace

## Abstract

**Introduction:**

The gut-liver axis has emerged as a focal point in chronic liver disorders, prompting more research into the role of the gut microbiota in liver cirrhosis. In individuals with liver cirrhosis, changes in the structure and function of the gut microbiota are closely tied to clinical prognosis. However, there is a scarcity of bibliometric evaluations conducted in this particular field.

**Methods:**

This study is aiming to conduct a complete analysis of the knowledge structure and centers pertaining to gut microbiota in liver cirrhosis using bibliometric methods. Publications on gut microbiota and liver cirrhosis from 2001 to 2023 are sourced from the Web of Science Core Collection. For the bibliometric analysis, we employ VOSviewer, CiteSpace, and the R package “bibliometrix”.

**Results:**

Our study encompasses a comprehensive collection of 3109 articles originating from 96 countries, with notable contributions from leading nations such as the United States and China. The quantity of publications concerning the gut microbiota of liver cirrhosis rises annually. The University of California San Diego, Virginia Commonwealth University, Zhejiang University are the primary research institutions. World Journal of Gastroenterology publishes the most papers in this field, while hepatology is the most frequently co-cited journal. These publications come from a total of 15,965 authors, and the most prolific authors are Bajaj Jasmohan S., Schnabl Bernd and Gillevet Patrick M., while the most co-cited authors are Bajaj Jasmohan S., Younossi Zobair M., and Reiner Wiest. In addition, “dysbiosis”, “gut microbiota”, “intestinal barrier”, “fecal microbiota transplantation”, and “complement-system” are the primary keywords of research trends in recent years.

**Discussion:**

This study offering a comprehensive insight into the research dynamics surrounding gut microbiota in patients with liver cirrhosis. It delineates the current research frontiers and hotspots, serving as a valuable guide for scholars.

## 1 Introduction

Liver cirrhosis is common worldwide and is a late manifestation of acute or chronic liver injury with multiple causes, such as excessive alcohol consumption, fatty liver, autoimmune diseases, cholestasis, and hepatitis B or C virus infections (Ginès et al., 2021). Under the long-term stimulation of pathogenic factors, hepatocytes repeatedly degenerate, become necrotic and eventually replaced by fibrotic tissue, which induces progressive deteriorates of liver function (Hernandez-Gea and Friedman, 2011; Zhou et al., 2014; Parola and Pinzani, 2019). Common complications of liver cirrhosis encompass portal hypertension, gastrointestinal bleeding, ascites, and hepatic encephalopathy (HE), among others (Garcia-Pagan et al., 2021). Individuals suffering from decompensated liver cirrhosis often have an unfavorable prognosis and usually need liver transplantation (Fouts et al., 2012; Shen et al., 2014; Engelmann et al., 2021). The mitigation of the burden caused by liver cirrhosis and its subsequent sequelae is a critical therapeutic concern for prompt resolution.

The human digestive system houses a diverse ecosystem teeming with bacteria, viruses, fungi, and other microbial entities, together referred to as the gut microbiome which is a result of the co-evolution of the gut microbiota with its host (Brody, 2020; Weersma et al., 2020). The constitution of the gut microbiota is easily affected by numerous factors such as age (Martino et al., 2022), drugs (Weersma et al., 2020) and diet (Gentile and Weir, 2018). Its dysregulation plays a pivotal role in the pathogenesis of chronic diseases including neurodegenerative diseases (Sampson et al., 2016), inflammatory bowel diseases (Franzosa et al., 2019) and cancer (Bai et al., 2022), particularly chronic liver disease (Frost et al., 2021). As the primary organ contacting microbial products that transverse the intestinal barrier, the liver is more susceptible to the alterations in the gastrointestinal microbiota in many ways (Tranah et al., 2021). Numerous prior investigations have highlighted the intricate interplay between gut microbiota and liver cirrhosis prognosis, noting that the balance of the gut microbiota is further disrupted with disease progression or decompensation (Bajaj et al., 2014b; Trebicka et al., 2021a). Therapeutic interventions concentrating on the gut microbiota, including probiotics, fecal microbiota transplantation (FMT), and antibiotics, have been extensively studied for chronic liver disease treatment, underscoring their therapeutic potential. Subsequent research has reinforced the importance of modulating the gut-liver axis (Bajaj, 2019). The preliminary studies on FMT in the treatment of decompensated cirrhosis demonstrate its safety profile and potential to improve patient prognosis while reducing hospitalizations, thereby paving the way for larger-scale investigations (Bajaj et al., 2017, 2019).

The utilization of bibliometrics as a research evaluation approach has become increasingly prevalent, employing a blend of quantitative and qualitative scientific analysis methodologies to investigate comprehensive knowledge about developmental trends within associated fields (Wallin, 2005). Bibliometric analysis is increasingly used to study various aspects of science (Ellegaard and Wallin, 2015). It can analyze the different features of publications in a particular field over a specific time period, including information and contribution about countries, institutions, authors, journals, keywords, references, etc. (Ke et al., 2020; Wu et al., 2022). Researchers can thus distill the current research landscape, identify hotspots, and discern progression trends in specific fields or diseases, offering valuable insights and guidance for future investigations (Lu et al., 2019). As a powerful tool for disciplinal development planning, bibliometrics has been drastically utilized in research across various medical fields, encompassing gynecological and obstetrical diseases (Brandt et al., 2019), pulmonary diseases (Lin et al., 2022), autoimmune diseases (Wu et al., 2022), cardiovascular diseases (Ma et al., 2021), among others. However, systematic bibliometric studies are scarce in the field of intestinal microbiology of liver cirrhosis. Our study is aiming to provide a comprehensive bibliometric analysis of the field of gut microbiota in liver cirrhosis, thus summarizing the present research status within this domain and anticipating further trends and prospects.

## 2 Materials and methods

### 2.1 Data search and retrieval strategy

For bibliometric studies, the Web of Science Core Collection (WoSCC) is an ideal source because of its extensive coverage of scholarly literature and the number of journals it indexes. All articles utilized in this study were sourced from the WoSCC database and retrieved on August 26, 2023. The results of literature retrieval are credible and authentic. The specific search strategy is as follows. The search formula is ((TS (topic) = (gut OR intestin* OR gastrointestin*) AND TS = (microbio* OR microflora OR flora OR bacteri* OR dysbiosis OR microecology OR 16Sr* OR metagenome)) OR TS = (prebiotic* OR probiotic* OR synbiotic*)) AND TS = (Liver cirrhosis OR hepatic cirrhosis OR liver fibrosis) AND LA (language) = (English). The publication type is limited to articles or reviews, and the time period of publication is from January 1, 2001, to August 26, 2023. We collated a total of 3,109 publications, comprising 2,004 articles and 1,105 reviews, for subsequent analysis.

### 2.2 Data process and visualization

VOS viewer (version 1.6.19) is a commonly used software designed for bibliometric analysis, boasting a robust graphical display capability. The tool allows users to construct and examine bibliometric maps of cooperation, co-citation, and co-occurrence networks for visual analysis. Additionally, it can extract critical information from a vast number of publications (van Eck and Waltman, 2010). Analysis of countries and institutions, analysis of journals and co-cited journals, analysis of authors and co-cited authors, analysis of co-cited references, and analysis of keyword co-occurrence were all performed mostly using the software in our research. Nodes on the map produced by VOS viewer provide details about a project, such as its location, organization, journal, and author. The size of the nodes signifies the total count of these items, and the various colors represent either the categories of the items or the years of their production. The line thickness connecting the nodes denotes the degree of collaboration or co-citation between items (Wu et al., 2021, 2022).

CiteSpace (version 6.2.4) is another bibliometric tool for citation visualization analysis (Synnestvedt et al., 2005). In our research, we utilized CiteSpace to examine journal coverage through a dual map approach and to analyze citations via Citation Bursts. With CiteSpace’s ability to identify and track the growth and shift of information, we can gain an intuitive understanding of cutting-edge developments, hotspots, and trends in these areas (Chen, 2004).

We employed the R package “bibliometrix” (version 4.1.3) from https://www.bibliometrix.org to analyze topic evolution and create a comprehensive publication network concerning gut microbiota in liver cirrhosis (Aria and Cuccurullo, 2017). The Journal Citation Reports 2022 was referenced to ascertain the journal’s impact factor (IF) and quartile. Furthermore, Microsoft Office Excel 2021 facilitated the analysis of the most prolific and highly cited countries/regions, journals, authors, institutions, co-cited references, and keywords throughout the articles.

## 3 Results

### 3.1 Quantitative analysis of annual publications

**Figure 1:**
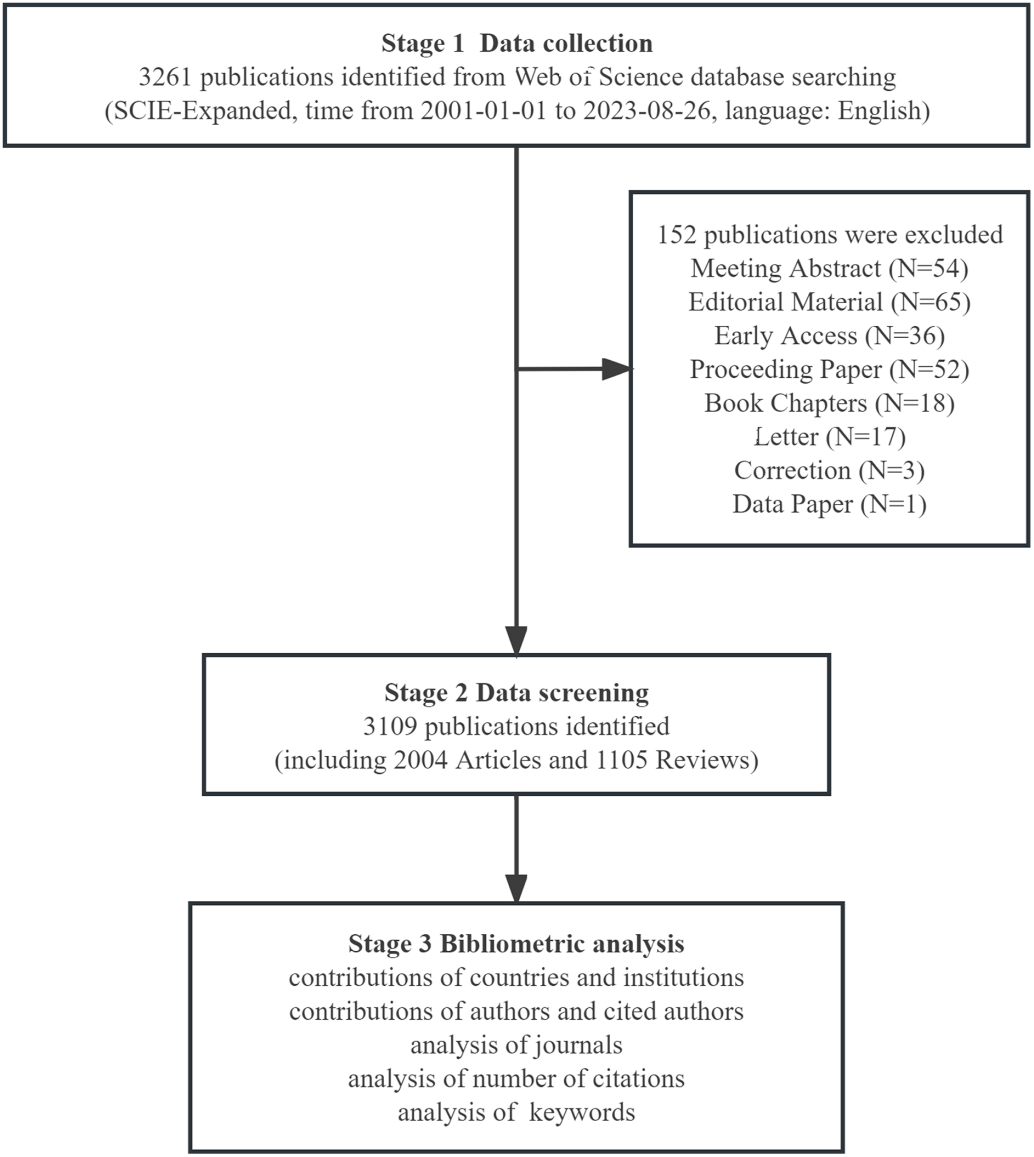
Flowchart of the publication selection.

**Figure 2:**
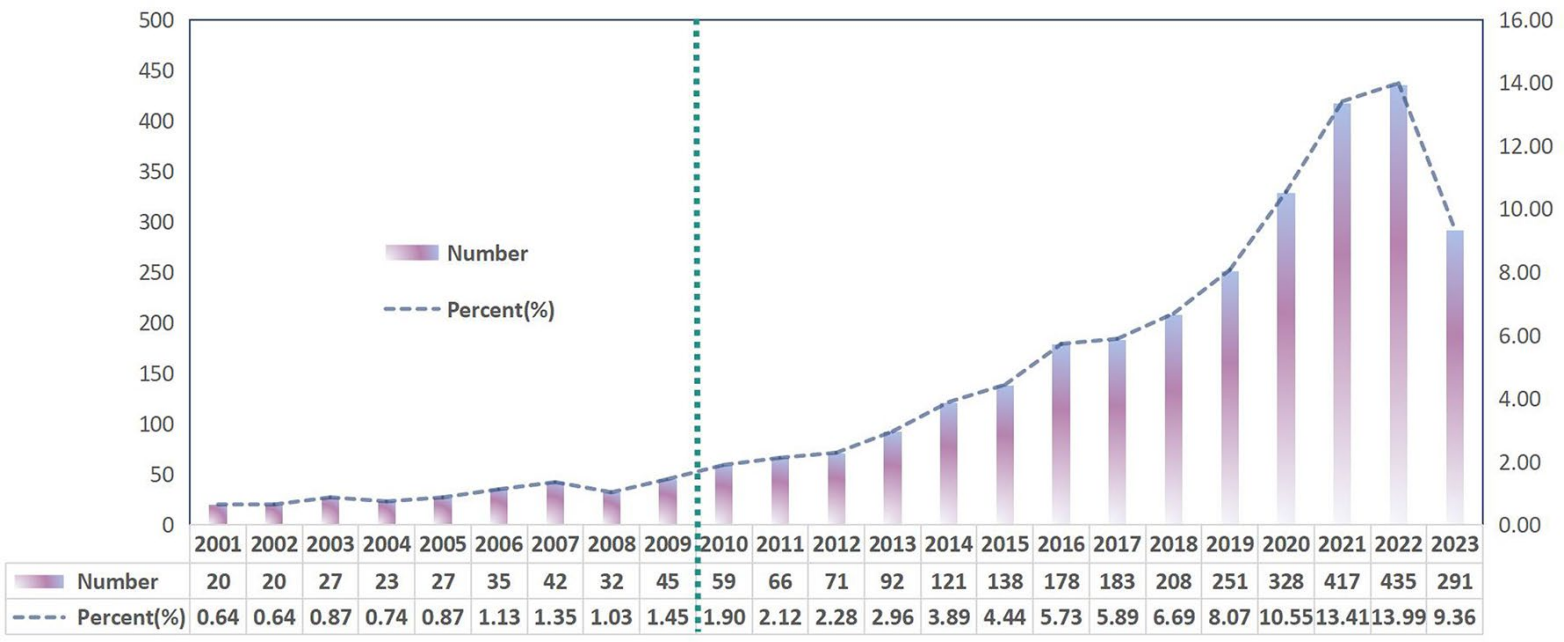
Annual number and trends of publications on gut microbiota in liver cirrhosis.

Based on our search criteria, 3,109 studies related to gut microbiota in liver cirrhosis were published in the past 23 years, comprising 2,004 articles and 1,105 reviews (Figure 1). Analyzing the annual publication growth rate and overall trend, we discerned two distinct phases: the initial phase (2001–2009) and the subsequent phase (2010–2023). As depicted in Figure 2, the initial phase, considered the research’s nascent stage, saw fewer than 50 publications annually, averaging 30.1 papers each year. In contrast, the subsequent phase recorded an average of 195.9 publications per year. In 2020, the number of publications was 328, which was 1.3 times more than that in 2019. In 2022, the average annual number of publications was approximately 435, and 291 papers have been published to date in 2023. Since 2017, the number of papers published on gut microbiota in relation to liver cirrhosis has increased substantially, particularly over the past 5 years.

### 3.2 Countries/regions and institution analyses

**Figure 3:**
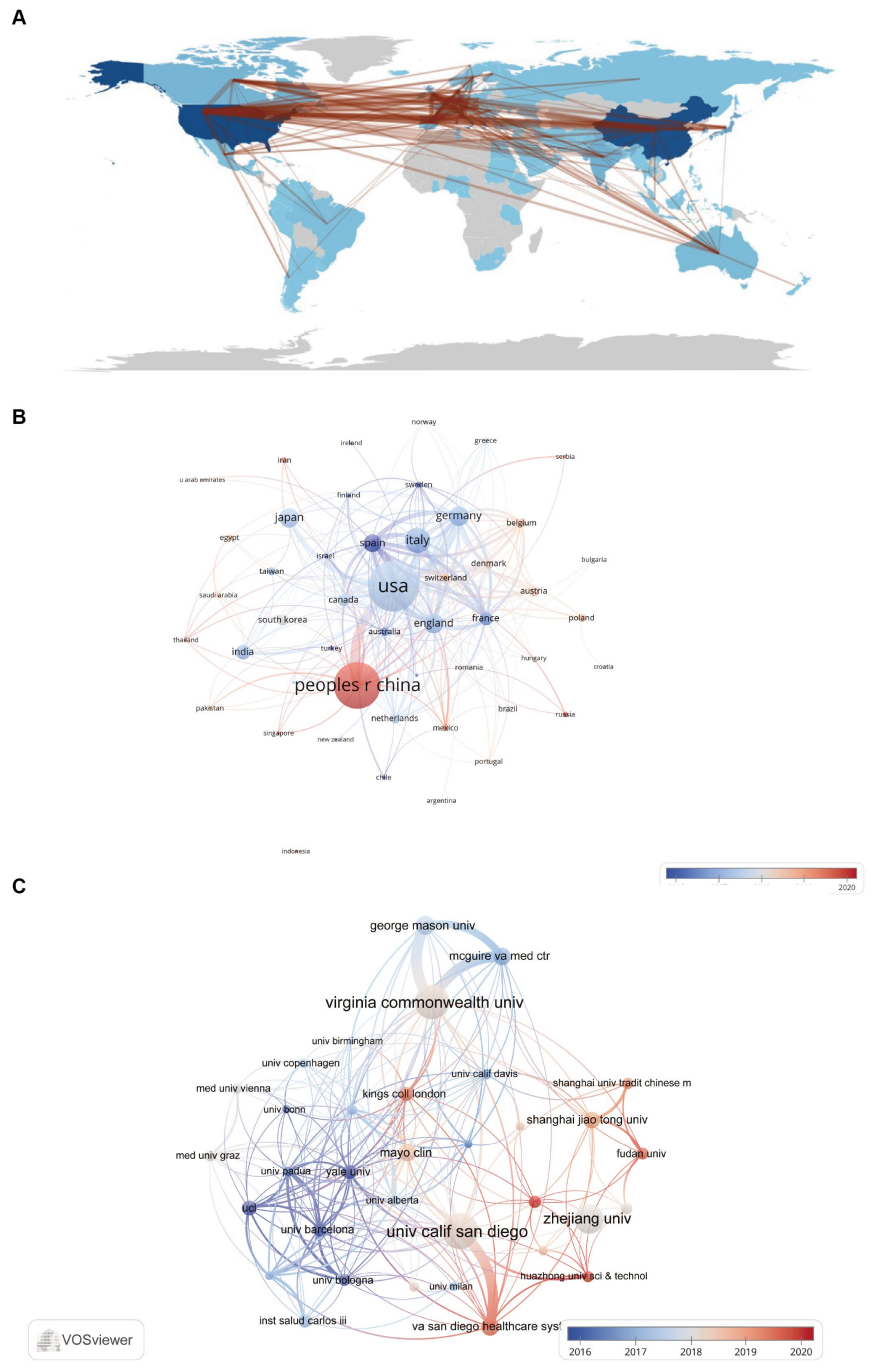
Visualization of countries/regions and institution contribution. **(A)** The geographical distribution of countries on research of gut microbiota in cirrhosis. **(B)** Visual depiction of countries involved in gut microbiota in cirrhosis studies. **(C)** Visual presentation of institutions on the research of gut microbiota in the context of cirrhosis.

The study of gut microbiota in liver cirrhosis has seen contributions from a total of 96 nations and regions, involving 3,437 institutions. The geographical breakdown of the top 10 nations was seen throughout Europe, Asia, and North America, with a predominant presence in Europe (*n* = 5) and Asia (*n* = 3) (Table 1). In this study, it was observed that the United States had the greatest number of published papers (*n* = 821, 19.68%) among the selected nations. China followed closely with a total of 726 papers (17.4%), while Italy and Germany had 296 (7.09%) and 206 (4.94%) articles, respectively. China and the United States together contribute roughly 37 percent of the overall amount. Following this, we conducted a screening process and using visual representation to analyze the data from 96 countries. Specifically, we focused on countries with a minimum of 5 publications, narrowing our analysis to a subset of 49 countries. This network is shown in Figures 3A,B. It is noteworthy to acknowledge the existence of several ongoing partnerships across various nations. For instance, while China has established partnerships with multiple countries, its strongest collaboration is with the United States. Similarly, the United States actively collaborates with countries including China, Germany, Spain, Canada, Japan, France, England, and Italy.

**Table 1:** Leading 10 countries on research of gut microbiota in cirrhosis.

Rank	Country	Counts
1	The United States (North America)	821 (19.68%)
2	China (Asia)	726 (17.40%)
3	Italy (Europe)	296 (7.09%)
4	Germany (Europe)	206 (4.94%)
5	Japan (Asia)	206 (4.94%)
6	England (Europe)	201 (4.82%)
7	Spain (Europe)	178 (4.27%)
8	India (Asia)	134 (3.21%)
9	France (Europe)	116 (2.78%)
10	Canada (North America)	106 (2.54%)

Table 2 reveals that among the top 10 institutions, three countries are represented. Notably, four of the top five institutions are in the United States, underscoring its dominant contribution to research in the realm of gut microbiota and liver cirrhosis. The four institutions that have published above 50 papers are: University of California San Diego (*n* = 111, 2.63%), Virginia Commonwealth University (*n* = 105, 2.49%), Zhejiang University (*n* = 86, 2.04%), and George Mason University (*n* = 52, 1.23%). Following this, we conducted a visual examination of 3,437 establishments, using a criterion of at least 20 publications as a threshold. This process yielded a final count of 37 institutions. Next, we constructed a collaboration network based on publication counts and inter-institutional ties. Figure 3C highlights that the University of California San Diego and Virginia Commonwealth University collaborate extensively with many other institutions, such as George Mason University, McGuire VA Medical Center and Virginia Commonwealth University, which work very closely with each other. Notably, the VA San Diego Healthcare System and the University of California San Diego also have a strong collaborative bond. In China, Shanghai University of Traditional Chinese Medicine, Fudan University and Shanghai Jiao Tong University are notably active in mutual cooperation. In addition, we also found that Zhejiang University began researching the field of gut microbiota in liver cirrhosis relatively early among several institutions with the highest publication volume in China. However, although Zhejiang University has published a lot of papers, it has seldom established close cooperation with other institutions.

**Table 2:** Leading 10 institutions on research of gut microbiota in cirrhosis.

Rank	Institution	Counts
1	University of California San Diego (The United States)	111 (2.63%)
2	Virginia Commonwealth University (The United States)	105 (2.49%)
3	Zhejiang University (China)	86 (2.04%)
4	George Mason University (The United States)	52 (1.23%)
5	Mayo Clinic (The United States)	49 (1.16%)
6	McGuire VA Medical Center (The United States)	47 (1.11%)
7	VA San Diego Healthcare System (The United States)	46 (1.09%)
8	Shanghai Jiao Tong University (China)	44 (1.04%)
9	University College London (England)	39 (0.92%)
10	Yale University (The United States)	37 (0.88%)

### 3.3 Journals and co-cited journals

**Figure 4:**
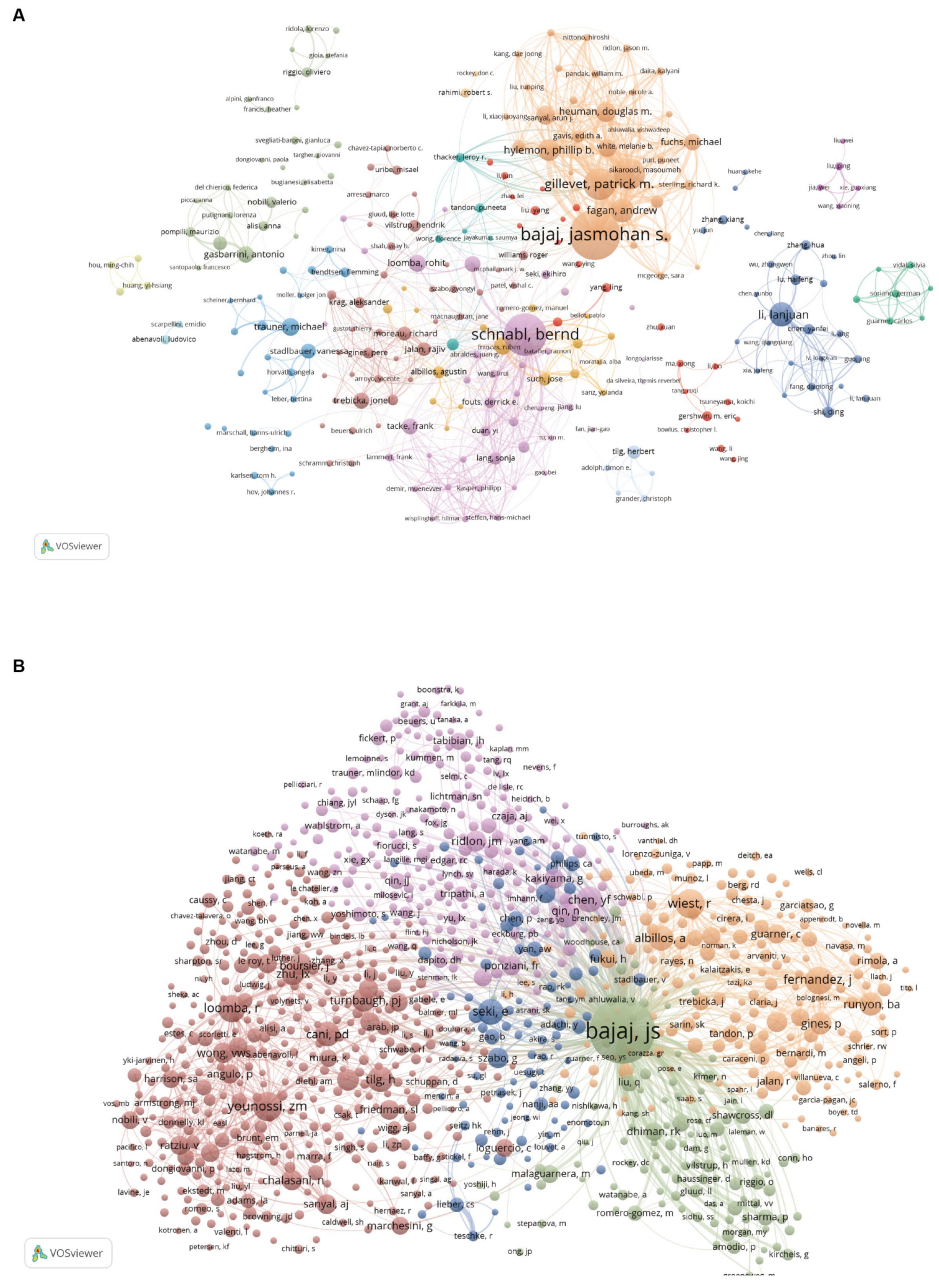
Visualization of authors and co-cited authors. **(A)** Visual representation of authors on the research of gut microbiota in the context of cirrhosis. **(B)** Visual presentation of co-cited authors on the research of gut microbiota in the context of cirrhosis.

A survey of journals and co-cited journals reveals that research articles on gut microbiota in liver cirrhosis spanned 825 academic journals. Notably, six journals each published over 50 articles on the topic. The *World Journal of Gastroenterology* led the list with 98 articles, followed closely by *Hepatology* and *Journal of Hepatology*, both with 81 articles, then the *International Journal of Molecular Sciences* (*n* = 77), *Liver International* (*n* = 59). Among the leading 15 journals, the *Journal of Hepatology* boasted the highest impact factor (IF) at 25.7, followed by *Hepatology* at 13.5. Figure 4 depicts the journal network constructed by selecting 41 journals with a minimum of 15 pertinent publications each. The Figure 4A illustrates active collaboration among these journals. The *Journal of Hepatology*, *World Journal of Gastroenterology*, *Hepatology* and *International Journal of Molecular Science* show a strong co-citation relationship respectively, constituting a quadrangular network. Additionally, *World Journal of Gastroenterology*, *International Journal of Molecular Science* and *Nutrients* has a positive co-citation relationship with each other.

Table 3 indicates that each of the top 15 most co-cited journals amassed over 1,500 citations, with three journals garnering more than 5,000 citations. *Hepatology* stands out as the most cited journal, with a total of 13,375 co-citations, highlighting its significant influence. This is trailed by the *Journal of Hepatology* (11,300 co-citations), *Gastroenterology* (5,181 co-citations), and *Nature Reviews Gastroenterology & Hepatology* (4,787 co-citations). Impressively, all the top 15 co-cited journals fall within either the first (Q1) or the second (Q2) quartiles of the journal ranking system. Of particular note, *Nature Reviews Gastroenterology* & *Hepatology* boasts the highest impact factor, with an IF of 65.1, succeeded by *Gastroenterology* (IF = 29.4) and *Journal of Hepatology* (IF = 25.7). The frequency with which a journal is cited serves as a significant metric for assessing its level of impact. In this research, we identified a total of 9,178 co-cited journals. After applying a screening process based on a minimum co-citation threshold of 250, a subset of 129 co-cited journals were selected to construct the co-citation network (Figure 4B). As depicted in Figure 4B, *Hepatology* shares a wide range of co-citation relationships with many journals such as *Journal of Hepatology*, *Gastroenterology*, *World Journal of Gastroenterology*, *Liver International*, *Clinical Gastroenterology* and *Hepatology*. In addition, it’s worth noting that three important journals including *Hepatology*, *Journal of Hepatology* and *Gastroenterology* constitute a remarkable co-citation network with each other.

**Table 3:** The leading 15 journals and co-cited journals for research of gut microbiota in cirrhosis.

Rank	Journal	Count	IF	JCR	Co-cited journal	Co-citation	IF	JCR
1	World Journal of Gastroenterology	98	4.3	Q2	Hepatology	13,375	13.5	Q1
2	Hepatology	81	13.5	Q1	Journal of Hepatology	11,300	25.7	Q1
3	Journal of Hepatology	81	25.7	Q1	Gastroenterology	5,181	29.4	Q1
4	International Journal of Molecular Sciences	77	5.6	Q1	Nature reviews Gastroenterology & Hepatology	4,787	65.1	Q1
5	Liver International	59	6.7	Q2	World Journal of Gastroenterology	4,196	4.3	Q2
6	Nutrients	57	5.9	Q1	Gut	3,789	24.5	Q1
7	Scientific Reports	48	4.6	Q1	American Journal of Physiology-Gastrointestinal and Liver Physiology	2,956	4.5	Q1
8	World Journal of Hepatology	42	2.4	None	Alimentary Pharmacology & Therapeutics	1993	7.6	Q1
9	European Journal of Gastroenterology & Hepatology	40	2.1	Q4	Metabolism-Clinical and Experimental	1826	9.8	Q1
10	PLoS One	40	3.7	Q2	International Journal of Molecular Sciences	1819	5.6	Q1
11	Digestive Diseases and Sciences	39	3.1	Q3	American Journal of Gastroenterology	1808	9.8	Q1
12	Journal of Gastroenterology and Hepatology	37	4.1	Q2	Journal of Gastroenterology and Hepatology	1749	4.1	Q2
13	Frontiers in Pharmacology	36	5.6	Q1	PLoS One	1,616	3.7	Q2
14	Alimentary Pharmacology & Therapeutics	33	7.6	Q1	Liver International	1,611	6.7	Q2
15	Frontiers in Immunology	33	7.3	Q1	Clinical Gastroenterology and Hepatology	1,531	12.6	Q1

Figure 4C provides a dual-map overlay, showcasing the citation connections between journals and their co-cited counterparts. This overlay illustrates the subject distribution of the journals related to gut microbiota in liver cirrhosis, allowing us to rapidly comprehend the frontiers and hotspots of each discipline and the flow of knowledge between disciplines (Shen et al., 2022). The right side of the diagram displays the clusters of journals that are referenced, while the left side indicates the clusters of journals that often cite other sources. The orange and green pathways illustrate dominant citation trends, suggesting that research from the Molecular/Biology/Genetics and Health/Nursing/Medicine domains predominantly receives citations from literature in the Molecular/Biology/Immunology and Medicine/Medical/Clinical sectors.

### 3.4 Authors and co-cited authors

**Figure 5:**
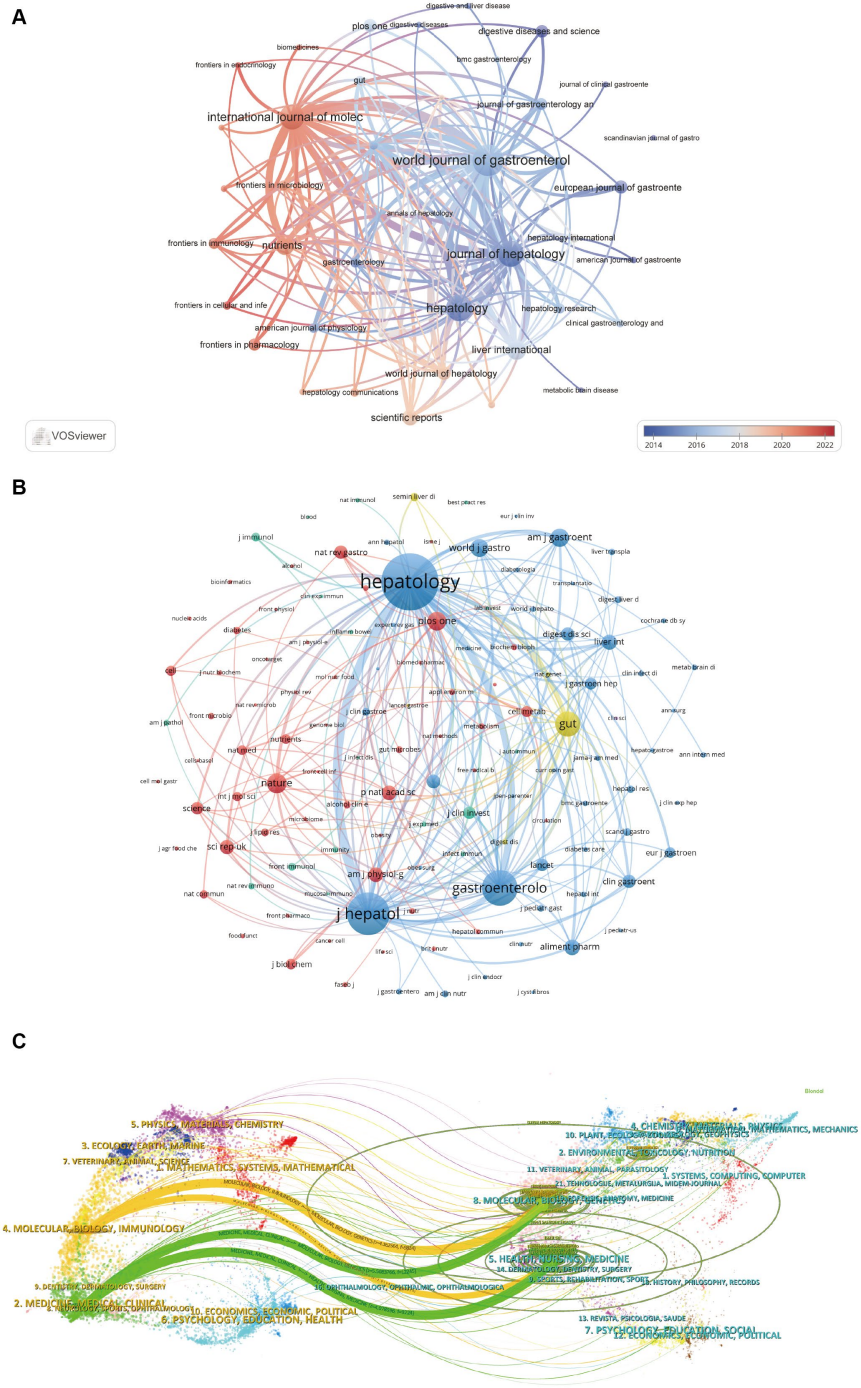
Visualization of journals and co-cited journals involvement. **(A)** Visual presentation of journals conducting research on gut microbiota in cirrhosis. **(B)** Visual presentation of co-citations among scholarly journals f on the research of gut microbiota in the context of cirrhosis. **(C)** The dual-map overlay of journals on research of gut microbiota in cirrhosis.

There are 15,965 authors contributing to the research. Table 4 summarizes the top 15 authors and co-citation authors according to the number of published papers and co-citation times, among which the top 15 authors all published at least 20 articles. Bajaj Jasmohan S. and Schnabl Bernd published the most papers, 82 and 66, respectively (Table 4), indicating that they have contributed to leading achievements in this field. We established a collaborative network comprised of authors who published at least 5 papers. Ultimately, 258 authors are finally shown in the picture (Figure 5A). Bajaj Jasmohan S., Schnabl Bernd, Gillevet Patrick M., Sikaroodi Masoumeh, and Lanjuan Li are represented by the largest nodes due to their most publications in this area. Furthermore, we observed intense cooperation between numerous authors. Bajaj Jasmohan S. closely collaborated with Gillevet Patrick M., Sikaroodi Masoumeh, and Fagan Andrew. Similarly, Schnabl Bernd worked closely with Lirui Wang, Fouts Derrick E., and Yi Duan. Lanjuan Li closely collaborated with Haifeng Lu and Yanfei Chen. However, it’s noteworthy that despite the substantial publications of Bajaj Jasmohan S., Schnabl Bernd, and Lanjuan Li, their direct and indirect collaboration with each other remains limited.

**Table 4:** The leading 15 authors and co-cited authors on research of gut microbiota in cirrhosis.

Rank	Authors	Count	Co-cited authors	Citations
1	Bajaj, Jasmohan S.	82	Bajaj, Jasmohan S.	2,838
2	Schnabl, Bernd	66	Younossi, Zobair M.	583
3	Gillevet, Patrick M.	46	Wiest, Reiner	580
4	Sikaroodi, Masoumeh	45	Fernández, Javier	534
5	Lanjuan Li	32	Loomba, Rohit	495
6	Fagan, Andrew	31	Seki, Ekihiro	461
7	Hylemon, Phillip B.	28	Yanfei Chen	454
8	Heuman, Douglas M.	24	Tilg, Herbert	448
9	Sanyal, Arun J.	23	Albillos, Agustín	438
10	White, Melanie B.	21	Cani, Patrice D.	407
11	Trauner, Michael	21	Nan Qin	384
12	Frances, Ruben	21	Turnbaugh, Peter J.	364
13	Yoshiji, Hitoshi	21	Runyon, Bruce A.	359
14	Suk, Ki Tae	21	Lixin Zhu	340
15	Loomba, Rohit	20	Boursier, Jérôme	325

A total of 66,339 co-cited authors were identified. After applying a screening criterion of having published 29 or more articles, a subset of 1,001 authors remained. Among these authors, it was observed that four specific authors were co-cited more than 500 occasions (Table 4). Bajaj Jasmohan S. is the most co-cited author (*n* = 2,838), demonstrating his significant impact on the research field and his essential role in promoting the field’s ongoing development. The following three were Younossi Zobair M. (*n* = 583), Wiest Reiner (*n* = 580) and Fernandez Javier (*n* = 534). The entire network of co-citations was depicted (Figure 5B). As observed in Figure 5B, active collaborations exist among the various co-cited authors.

### 3.5 Co-cited references

**Figure 6:**
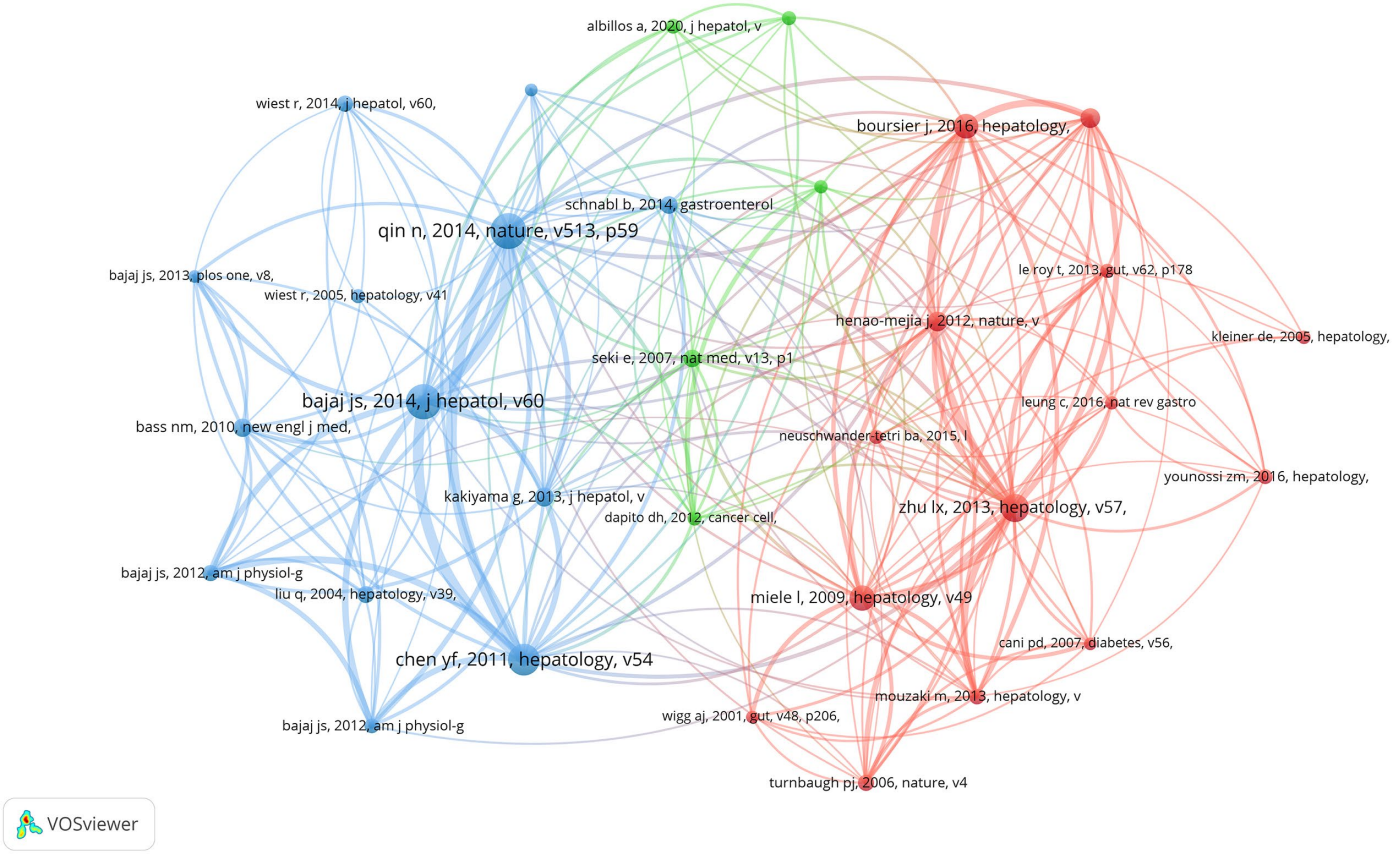
Visual presentation of references among authors on the research of gut microbiota in the context of cirrhosis.

In the preceding time period, an overall total of 107,643 co-cited references have been documented in the realm of gut microbiota research pertaining to cirrhosis. Each of the co-cited references enumerated in Table 5 has received a minimum of 194 citations, with three of them being co-cited in excess of 300 times. We have chosen references that have been mentioned 130 times or more in order to create a network of co-cited literature. A total of 32 co-cited references were obtained (Figure 6). As depicted in Figure 6, the article “Qin N., 2014, Nature” is an important article with the highest citation frequency and significant influence in this field. This article demonstrates a strong co-citation relationship with “Bajaj J. S., 2014, J. Hepatol.,” “Chen Y. F., 2011, Hepatology,” and” Kakiyama G., 2013, J. Hepatol. “among others.

**Table 5:** The leading 10 co-cited references contributing to the research of gut microbiota in cirrhosis.

Rank	Co-cited reference	Citations
1	Shen et al. (2014)	379
2	Bajaj et al. (2014b)	371
3	Chen et al. (2011)	335
4	Zhu et al. (2013)	298
5	Miele et al. (2009)	264
6	Boursier et al. (2016)	257
7	Loomba et al. (2017)	209
8	Henao-Mejia et al. (2012)	205
9	Kakiyama et al. (2013)	198
10	Bass et al. (2010)	194

### 3.6 Reference with citation bursts

**Figure 7:**
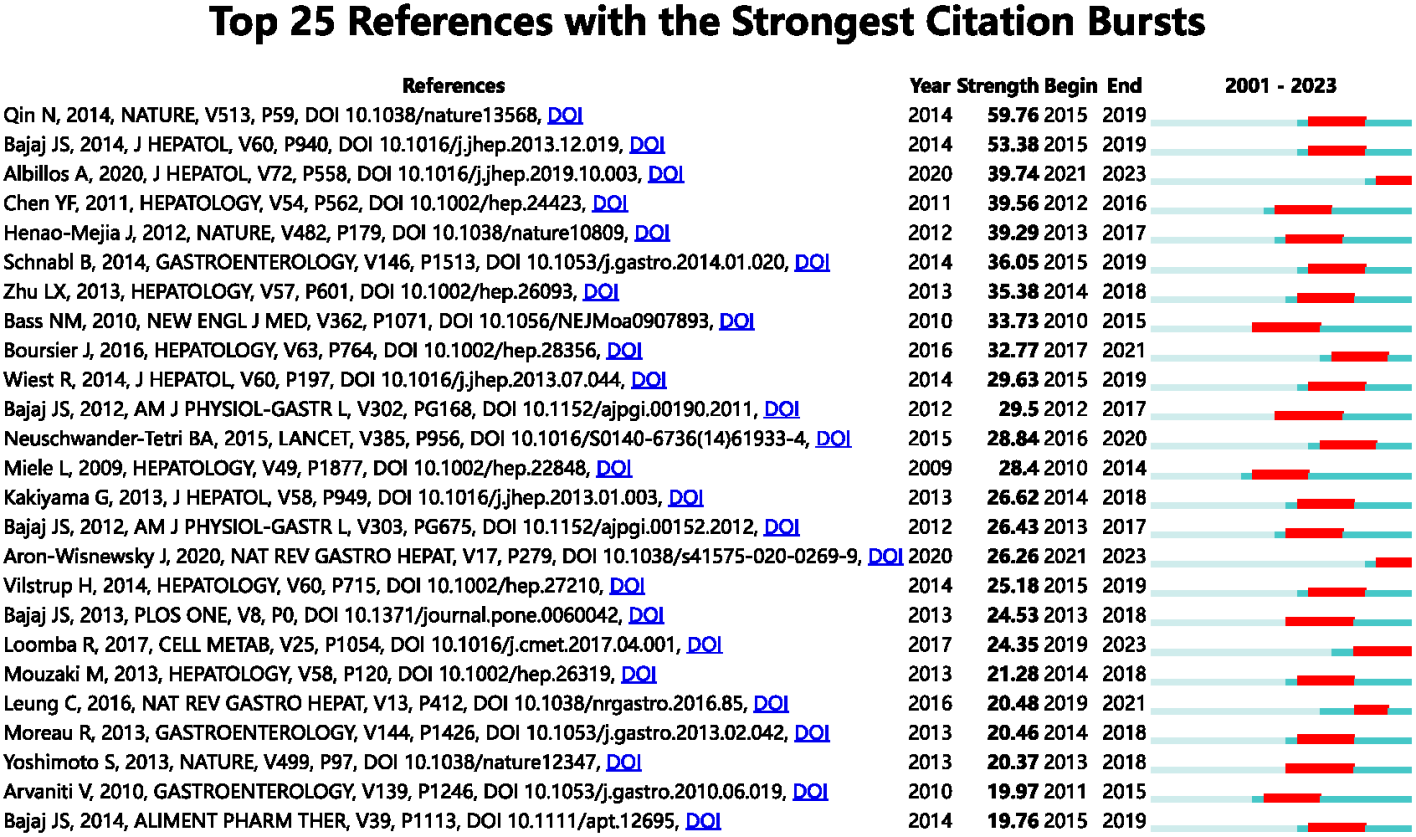
The leading 25 references with citation bursts are depicted. A red bar signifies an elevated count of citations for that specific year.

The term “Reference with Citation Bursts” refers to scholarly papers that have been extensively cited within a specific timeframe. In the present investigation, a total of 25 references exhibiting citation bursts were found using the CiteSpace software (Figure 7). As seen in Figure 7, each bar corresponds to a single year, with the red bar indicating a period of significant citation burst (Huang et al., 2019). The earliest citation bursts for references appeared in 2010 and the latest in 2021. The article titled “Alterations of the human gut microbiome in liver cirrhosis” by Qin et al., published in Nature, had the highest citation strength (strength = 59.76) among the references. Notably, this publication saw a significant increase in citations between 2015 and 2019. The article titled “Altered profile of human gut microbiome is associated with cirrhosis and its complications” published in the Journal of Hepatology in 2014 by Bajaj JS et al. had the second highest citation burst strength (53.38). The citation burst period for this article extended from 2015 to 2019. The three references, namely “The gut-liver axis in liver disease: pathophysiological basis for therapy,” “Gut microbiota and human NAFLD: disentangling microbial signatures from metabolic disorders,” and “Gut Microbiome-Based Metagenomic Signature for Non-invasive Detection of Advanced Fibrosis in Human Nonalcoholic Fatty Liver Disease,” all continue to be a subject of significant research interest in 2023. In general, the set of 25 references exhibits a burst strength range spanning from 19.76 to 59.76, with an endurance strength varying between 2 and 5 years. Table 6 provides a comprehensive summary of the primary research topics discussed in the 25 references, arranged according to the literature order shown in Figure 7.

**Table 6:** The primary scientific discoveries of the 25 references with strong citations bursts.

Rank	Strength	Main research content
1	59.76	Identified alterations of the human gastrointestinal microbiome in liver cirrhosis (Shen et al., 2014)
2	53.38	Cirrhosis is accompanied by progressive alterations in the gastrointestinal microbiome, which become more severe during decompensation (Bajaj et al., 2014b)
3	39.74	The gut-liver axis is the pathophysiological premise for gut-centered therapy in liver disease (Albillos et al., 2020)
4	39.56	Patients with cirrhosis have different fecal microbial communities compared to healthy individuals (Chen et al., 2011)
5	39.29	Dysbiosis mediated by inflammasomes controls the progression of NAFLD and adiposity (Henao-Mejia et al., 2012)
6	36.05	The combination of liver injury and intestinal dysbiosis contributes to the development of liver disease (Schnabl and Brenner, 2014)
7	35.38	Nonalcoholic steatohepatitis patients’ gut microbiomes were characterized (Zhu et al., 2013)
8	33.73	Rifaximin substantially decreased the risk of hepatic encephalopathy in cirrhotic patients (Bass et al., 2010)
9	32.77	Gut dysbiosis and a change in the gut microbiota’s metabolic function are associated with the severity of nonalcoholic fatty liver disease (Boursier et al., 2016)
10	29.63	Pathological bacterial translocation in liver cirrhosis (Wiest et al., 2014)
11	29.5	The gut microbiome is dramatically altered in patients with hepatic encephalopathy and is associated with cognition (Bajaj et al., 2012b)
12	28.84	This study investigates the effectiveness of obeticholic acid in treating non-alcoholic steatohepatitis in adult patients (Neuschwander-Tetri et al., 2015)
13	28.4	The disruption of intercellular tight junctions in the intestines may lead to an increase in intestinal permeability, which may have a substantial effect on the development of hepatic fat deposition (Miele et al., 2009)
14	26.62	The gut microbiota’s influence on the fecal bile acid composition in individuals with cirrhosis (Kakiyama et al., 2013)
15	26.43	In cirrhosis and hepatic encephalopathy, the colonic mucosal microbiome is distinct from the stool microbiome and is associated with cognition and inflammation (Bajaj et al., 2012a)
16	26.26	A comprehensive analysis of microbiome characteristics for human NAFLD (Aron-Wisnewsky et al., 2020)
17	25.18	Practice guidelines for hepatic encephalopathy published in 2014 (Vilstrup et al., 2014)
18	24.53	Rifaximin modulates the metabiome in patients with cirrhosis and mild hepatic encephalopathy (Bajaj et al., 2013)
19	24.35	A microbiome panel based on the metagenome can accurately diagnose advanced fibrosis (Loomba et al., 2017)
20	21.28	NAFLD development may be influenced by intestinal microbiota (Mouzaki et al., 2013)
21	20.48	The central role of the microbiome in NAFLD (Leung et al., 2016)
22	20.46	Identified the diagnostic criteria of acute-on-chronic liver failure and described the development of this syndrome in European patients with acute decompensation (Moreau et al., 2013)
23	20.37	Through the senescence secretome, an obesity-induced intestinal microbial metabolite boosts liver cancer (Yoshimoto et al., 2013)
24	19.97	Infections quadruple the mortality rate in cirrhotic patients (Arvaniti et al., 2010)
25	19.76	The impact of *Lactobacillus* GG on the gut microbiota, metabolome, and endotoxemia in individuals diagnosed with cirrhosis is being investigated (Bajaj et al., 2014a)

### 3.7 Keywords co-occurrence analysis

The use of keyword co-occurrence analysis enables the expeditious identification of prominent areas of study focus within a certain academic domain. This analysis can also highlight the most essential topics and forecast potential future research directions. Table 7 displays the top 20 high-frequency keywords. Among them, cirrhosis, gut microbiota, and liver cirrhosis each occurred more than 200 times, representing the main research directions of liver cirrhosis-related gut microbiota.

**Table 7:** Leading 20 keywords in the research on the gut microbiota in relation to cirrhosis and 10 keywords related to bacterial taxa.

Rank	Keyword	Counts	Total link strength
1	Non-alcoholic fatty liver disease	446	950
2	Cirrhosis	404	832
3	Gut microbiota	339	645
4	Liver cirrhosis	264	439
5	Non-alcoholic steatohepatitis	252	599
6	Probiotics	195	518
7	Inflammation	191	476
8	Microbiome	179	434
9	Hepatic encephalopathy	177	374
10	Microbiota	168	421
11	Bacterial translocation	142	336
12	Gut-liver axis	120	337
13	Fibrosis	118	284
14	Liver fibrosis	118	188
15	Dysbiosis	114	310
16	Hepatocellular carcinoma	107	246
17	Liver disease	106	190
18	Portal hypertension	95	209
19	Bile acids	91	218
20	Gut microbiome	84	188

Rank	Keyword (taxa)	Counts	Total link strength
1	*Lactobacillus*	49	22
2	*Escherichia coli*	43	17
3	*Helicobacter pylori*	32	7
4	*Akkermansia muciniphila*	31	8
5	*Clostridium difficile*	20	5
6	*Clostridium difficile* infection	20	5
7	*Helicobacter pylori* infection	15	5
8	*Bifidobacterium*	14	12
9	*Lactobacillus acidophilus*	10	3

**Figure 8:**
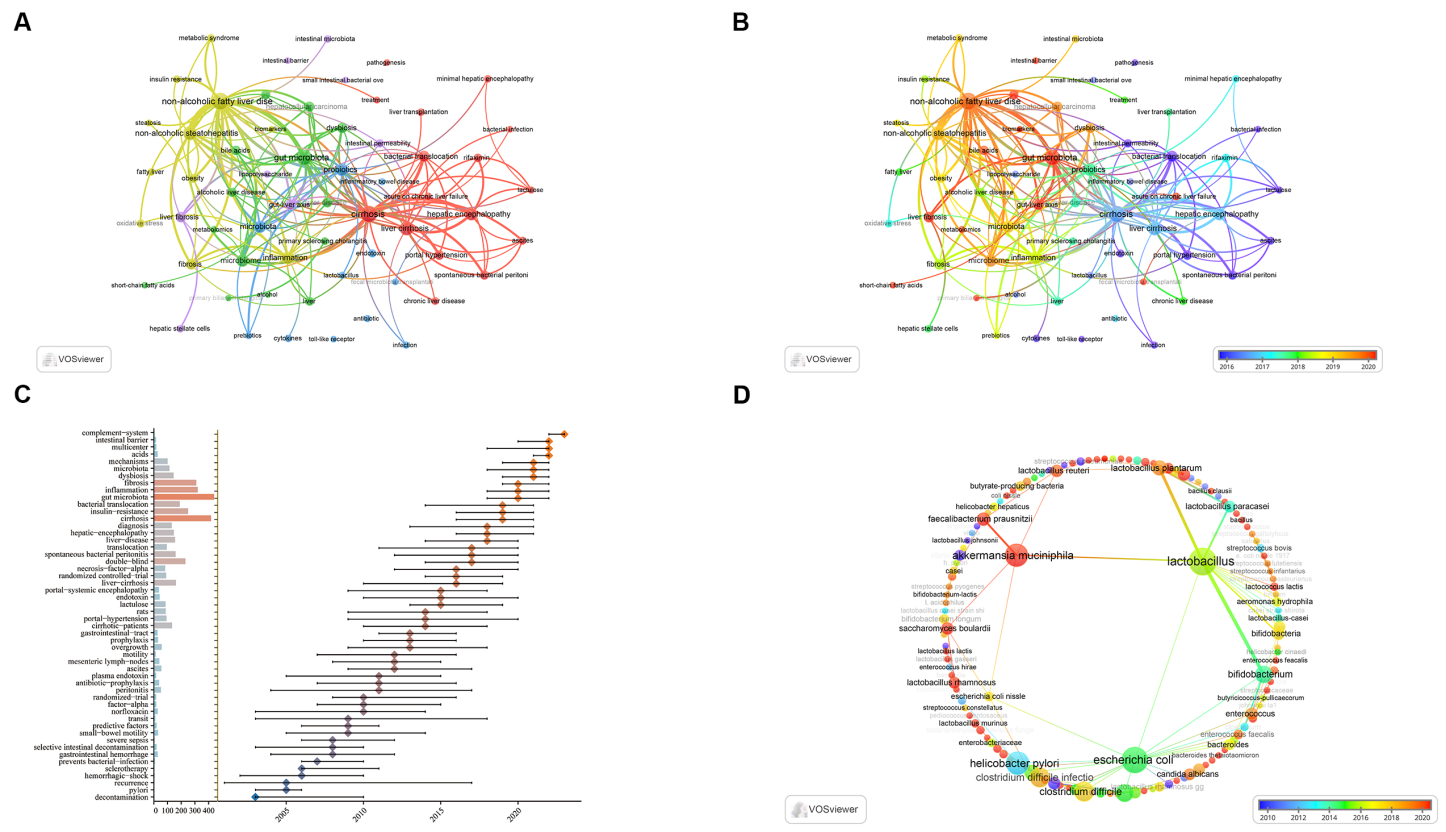
Visualization of keywords and trend topics. **(A)** Keyword cluster analysis. **(B)** Overlay visualization analysis revealed the evolution of co-occurring keywords over time. **(C)** The trend topic analysis of the keywords. **(D)** Overlay visualization analysis revealed the evolution of co-occurring keywords related to bacterial taxa over time.

As shown in Figure 8A, the keywords with the highest frequencies are non-alcoholic fatty liver disease (NAFLD) (446 times), cirrhosis (404 times), and gut microbiota (339 times). A total of five clusters were found, each reflecting a distinct study path. Additionally, the evolution of keywords over time is shown in the overlay representation depicted in Figure 8B. Previous keywords are shown in purple, while red represents the most recent keywords. TLR, bacterial translocation, lactulose, endotoxin, intestinal permeability, and cytokines were the main themes of early studies. In contrast, we can see that red frames are popular keywords in recent years, which include gut-liver axis, metabolomics, short-chain fatty acids (SCFAs), bile acids and FMT. The results of these analyses can help researchers speculate on future research directions and progress in the field of gut microbiota in liver cirrhosis.

Trend topic Analysis of the keywords (Figure 8C) shows that as early as 2005, the main keywords are represented by selective intestinal decontamination, transit, norfloxacin. This indicates that researchers began to study intestinal decontamination therapy and the role of intestinal bacteria in liver cirrhosis and its related complications. The main keywords between 2005 and 2015 were lactulose, bacterial translocation, endotoxin, portal-systemic encephalopathy, ascites. There is no doubt that researchers have begun to actively explore the treatment of liver cirrhosis by regulating intestinal bacteria in order to reduce the incidence of related complications. Until 2018, focuses were shifted towards dysbiosis, gut microbiota, intestinal barrier, mechanisms and so on, which indicates that the aim of researches has changed from simple sterilization towards improving the balance of gut microbiota. In 2023, complement-system is likely to exhibit research potential in the future.

Of note, although bacterial taxon is a critical topic in the study of gut microbiota, the number of keywords occurrence in this topic seems insufficient for visualization. As a result, the routine co-occurrence analysis (Figure 8A) is technically impossible to specifically demonstrate keywords related to bacterial taxa. To reveal the trend and pattern of this topic, we manually pick up related keywords from the whole keywords pool, demonstrating an independent overlay visualization of co-occurring keywords related to bacterial taxa over time (Figure 8D). Table 7 shows the leading 10 keywords related to bacterial taxa. *Lactobacillus* (49 times), *Escherichia coli* (43 times) and *Helicobacter pylori* (32 times) are the most frequent keywords. In addition, *Lactobacillus* and *Escherichia coli* show a wide correlation with other keywords. These bacterial taxa have gained major concern and exhibited critical value in the research area of liver cirrhosis.

## 4 Discussion

The notion of the gut-liver axis, which emerged some decades ago, has shed light on the significant involvement of gut microbiota in chronic liver illnesses. This revelation has opened up new avenues for investigating prospective treatment strategies towards different liver diseases such as alcoholic liver disease (Duan et al., 2019), NAFLD (Lei et al., 2022) and liver cancer (Ma et al., 2018). Fruitful works have indicated a wide range of novel insights into the gut microbiota in liver cirrhosis (Trebicka et al., 2021a,b). We utilized the WoSCC database to analyze 3,109 articles over the last decades in this field. From 2001 to 2009, the annual number of published papers was less than 50 and experienced a slow but steady development, manifesting that the research on the gut microbiota of liver cirrhosis was in its infancy during this period. After 2010, there was a notable surge in the quantity of published articles, with an average yearly figure of 195.9. Furthermore, the current data suggests a continuous upward trend of the ongoing study in this particular domain, indicating great scientific interest and focus.

The results pertaining to country distribution reveal that the United States, China, and Italy are the top three countries for studying gut microbiota in relation to liver cirrhosis, with the United States ranking first. We have observed that the United States cooperates actively with numerous nations, most closely with China. Regarding research institutions, the top 10 are spread across three countries. Remarkably, four-fifths of these are based in the United States, and several of these institutions maintain strong collaborative ties. For instance, George Mason University, McGuire VA Medical Center, and Virginia Commonwealth University work closely together. Three institutions in China collaborate actively, namely Shanghai Jiao Tong University, Fudan University, and Shanghai University of Traditional Chinese Medicine. Additionally, there is also active collaboration between Huazhong University of Science and Technology, the University of California San Diego, and the VA San Diego Healthcare System. Nevertheless, it’s worth mentioning that despite Zhejiang University’s significant publication output, its limited collaboration with other universities might potentially impede the sustained progress of academic research. Although several nations have established cooperative partnerships, there is a need for further enhancement in the scope and depth of collaboration among specialized organizations. It is very advisable to foster extensive collaboration and facilitate exchanges among research institutes across different nations in order to collectively advance the academic progress.

The *World Journal of Gastroenterology* leads in publications on gut microbiota in liver cirrhosis, with 98 articles, marking its significance in the field. Of the top 15 journals, *Journal of Hepatology* boasts the highest impact factor (IF = 25.7, Q1), followed by *Hepatology* (IF = 13.5, Q1). Most of the top co-cited journals are renowned Q1 journals, with six possessing an IF above 10, underscoring their pivotal role in the field. Current research primarily appears in Molecular/Biology/Immunology and Medicine/Medical/Clinical journals, citing work from Molecule/Biology/Genetics or Health/Nursing/Medicine realms. This reflects a comprehensive approach to research, bridging basic studies with clinical applications.

Among the top 10 authors in this field, the average number of publications per author exceeds 20 papers, with Bajaj Jasmohan S., Schnabl Bernd, and Gillevet Patrick M. publishing more than 40 papers each. Professor Bajaj Jasmohan S. has published the most papers for 82. Bajaj Jasmohan S. and his team have dedicated themselves to this field and their researches have played a vital role in the advancement of this discipline. The researchers made the observation that the gut microbiome undergoes a gradual transformation as cirrhosis advances. They proposed that the “cirrhosis dysbiosis ratio” offers a significant quantitative metric to delineate changes in the gut microbiome as cirrhosis progresses (Bajaj et al., 2014b). In 2013, they revealed a complex relationship between bile acid levels and the reciprocal regulation of intestinal microbiota in cirrhosis (Ridlon et al., 2013). In the same year, further studies investigated the relationship between the concentration of fecal bile acid and the intestinal microbiome in cirrhotic patients of varying severity. Alterations in fecal bile acid profiles in individuals with cirrhosis are influenced by pivotal microorganisms within the gut microbiota (Kakiyama et al., 2013). Bajaj Jasmohan S. and Gillevet Patrick M., upon analyzing the gut microbiome in patients with liver cirrhosis, identified a correlation between the concentrations of gut microbiota-derived metabolites in the bloodstream and the incidence of acute-on-chronic liver failure (ACLF) as well as mortality rates in these patients. The use of this biomarker has the potential to effectively and promptly identify individuals at a heightened risk of ACLF and mortality (Bajaj et al., 2020). In conclusion, their works provide a cornerstone for related studies which establish solid bridge between the pattern of microbial agents and the progression of liver cirrhosis.

In 2021, they also investigated the influence of antibiotic resistance genes (ARGs) burden in intestinal metagenomes on disease progression and prognosis. They discovered that the intestinal ARGs burden was higher in cirrhotic patients than in controls and worsened with disease progression. Nonetheless, rifaximin has a good regulatory effect on ARGs (Shamsaddini et al., 2021). Bajaj Jasmohan S. has also studied the connection between gut microbiota and inflammatory response, demonstrating that gut microbiota can promote the development of neuroinflammatory and systemic inflammatory responses in cirrhotic mice (Kang et al., 2016; Liu et al., 2020). Later, his research on gut microbiota expanded the understanding of fungi, revealing the presence of a significant fungal dysbiosis in cirrhotic patients. The ratio of Bacteroidetes to Ascomycota can independently forecast 90 days hospitalization outcomes in patients with cirrhosis (Bajaj et al., 2018b). Furthermore, the authors offered a succinct review of ongoing research exploring the gut microbiota as a possible therapeutic focus on liver illness. This includes the investigation of probiotics, FMT, and precision medicine focused on particular microorganisms (Bajaj et al., 2022). The researchers conducted an experimental study to validate the reduction in abundance of ARGs in the gut microbiota of patients with decompensated cirrhosis after FMT. They further noted that FMT could potentially reverse the damage caused by antibiotics to the constitution and functionality of the gut microbiota in individuals with cirrhosis (Bajaj et al., 2018a, 2021). On the one hand, their recent works put forward the understanding of the relationship between gut microbiota (including fungi) and ARGS as well as inflammation in liver cirrhosis. On the other hand, they also made extensive explore on the therapeutic practice of microbial intervention towards liver cirrhosis.

Among the Chinese authors, Lanjuan Li has published most papers, reaching 32. As early as 2011, she revealed the features of the intestinal microflora in cirrhotic patients (Chen et al., 2011). By 2014, she delved deeper into the changes in gut microbiota in individuals with liver cirrhosis, constructed a gene catalog of the intestinal microbiome, and selected 15 optimal biomarkers with high specificity for distinguishing liver cirrhosis patients from healthy individuals (Shen et al., 2014). Moreover, she also disclosed the involvement of gut microbiota, bile acids, and the T helper cell 17/Interleukin 6 axis in the progression of liver fibrosis associated with hepatitis B (Chen et al., 2020). These studies also made important contribution on the microbial aspects of prognosis and pathogenesis in liver cirrhosis.

The author with the most co-citations is Bajaj Jasmohan S. (*n* = 2,838), followed by Younossi Zobair M. (*n* = 583), Wiest Reiner (*n* = 580), and Fernandez Javier (*n* = 538). The fact that Bajaj Jasmohan S. has the most published articles and is the most cited author is sufficient evidence that he facilitates the development of intestinal microbiota in liver cirrhosis. Bajaj Jasmohan S. is dedicated to studying not only the alterations in intestinal flora in liver cirrhosis, but also the effects of intestinal microbiota-derived metabolites in serum and ARGs in the gut metagenome on the progression and prognosis of liver cirrhosis. Jasmohan S. Bajaj and his team, through their clinical research, have substantiated that FMT can improve the outcomes for patients suffering from liver cirrhosis. Additionally, their investigations expanded into the realm of mycobiome, pinpointing an imbalance in fungal populations within the intestines of patients with cirrhosis.

Younossi Zobair M. has primarily centered his research on investigating advanced fibrosis and cirrhosis in patients diagnosed with NAFLD (Younossi et al., 2018, 2019). Early in 2014, Wiest Reiner summarized the issue of pathological bacterial translocation (PBT) in patients with liver cirrhosis. Liver cirrhosis can significant damage the intestinal endothelial and muco-epithelial barriers, resulting in PBT into the portal vein system. Farnesoid × receptor agonists can decrease PBT (Wiest et al., 2014; Sorribas et al., 2019). These studies have laid the foundation for the future therapies of liver cirrhosis by targeting the intestinal microbiota.

A co-citation relationship is established when multiple papers are jointly cited by one or more subsequent publications. Consequently, co-cited reference is an important method of analysis in bibliometrics research, and citation frequency can reflect an article’s influence in a particular research field (Sun et al., 2022). Highly cited papers are typically of high quality, innovative, and make a substantial contribution to their field, which is the basis for promoting the continuous development of the research field. Table 4 displays the 10 most frequently co-cited papers. These studies have been co-cited a minimum of 194 times, with the first three being cited more than 300 times, which have significant impacts on this field. Lanjuan Li and Bajaj JS have each authored two publications that are ranked inside the top 10 most frequently referenced papers. The paper titled “Alterations of the human gut microbiome in liver cirrhosis” authored by Lanjuan Li in 2014, as published in *Nature*, has garnered a total of 379 citations, so establishing itself as the most often referenced work within the domain of liver cirrhosis research. The researchers have disclosed distinct modifications in the gut microbiota among persons with cirrhosis and have found gene clusters that exhibit varying levels of abundance between cirrhotic patients and those who are in good health, as determined using quantitative metagenomic analysis (Shen et al., 2014). In 2011, Lanjuan Li released a work that has been mentioned 335 times, which examined the attributes of the intestinal microbial population in individuals diagnosed with liver cirrhosis. The proliferation of Enterobacteriaceae, Streptococcaceae, and other potentially dangerous bacteria, along with the decline in helpful bacteria like Lachnospiraceae, is expected to have implications for the prognosis of individuals diagnosed with liver cirrhosis. The study titled “Altered profile of the human gut microbiome in association with cirrhosis and its complications,” authored by Bajaj, Jasmohan S., was published in the esteemed *Journal of Hepatology*. This research paper garnered significant attention, being cited 371 times, making it the second most frequently referenced publication. The study findings revealed a progressive alteration in the gut microbiome imbalance as cirrhosis advanced, offering valuable insights into the relationship between cirrhosis and the human gut microbiome in the year 2014 (Bajaj et al., 2014b). Another study published by Bajaj Jasmohan S., cited 198 times, confirmed that the changes in fecal bile acid patterns in cirrhotic patients were regulated by key microorganisms in the gut microbiota (Kakiyama et al., 2013). These masterworks are in crucial role in the establishment of the connection between gut microbiota and liver cirrhosis. They collectively provided valuable and detailed evidences covering static status and dynamic trends in this connection, which constructed the academic frame and provided inspiration for further studies.

References characterized by citation bursts are those that experience frequent and vigorous discussion or citation by academics within a specific timeframe. The burst detection technique is designed to identify and record instances of significant growth in the popularity of citations within a certain timeframe. The analysis of research hotspots in an academic subject is a valuable approach for investigating the growth of scholarly interests. This method is useful in efficiently identifying prominent areas of study or specific themes within a given field. The analysis conducted revealed that the first occurrence of a surge in reference citations within the field started in 2010 and persisted until 2023. There are two studies published in 2010, one of which was researched by Bass N. M. in 2010, demonstrating that rifaximin, as a minimally absorbed antibiotic, can effectively reduce the risk of HE in patients with cirrhosis (Bass et al., 2010). Another notable study, conducted by Miele L. in 2009, underscored the notion that elevated intestinal permeability, brought about by the disruption of tight junctions amidst intestinal cells, could play a pivotal role in the onset of liver fat accumulation (Miele et al., 2009). Both of these studies set the stage for a subsequent series of studies. As shown in Figure 7, most of the burst citations are still in progress. The most recent study started in 2021 and is still in burst citation status until 2023. Among the top 25 references exhibiting significant citation bursts, the research by Qin et al. in 2014, which delved into the changes in the functionality and composition of the gut microbiota in liver cirrhosis patients, stood out with the highest burst strength value. Using quantitative metagenomic analysis, they established a cohort reference gene set of 2.69 million genes, of which 36.1% were novel genes. In addition, they identified 66 gene clusters whose abundance varied between cirrhosis patients and healthy individuals (Shen et al., 2014). This finding has a profound influence on the gut microbiota of liver cirrhosis and lays the foundation for future research in this field.

Keyword co-occurrence analysis is a widely used method in the field of bibliometrics to find significant research themes. The analysis may serve as a valuable tool for tracking the progression of scholarly interests and gaining deeper insights into prominent areas of investigation (Pei et al., 2022). We performed overlay visualization analysis and trend topic analysis of keywords in the field. As shown in Figure 8A, the keywords that frequently appeared in the red cluster were cirrhosis, liver cirrhosis, bacterial translocation, HE, portal hypertension, ascites, lactulose, rifaximin. Numerous prior investigations have substantiated the presence of intestinal microecological dysbiosis in individuals diagnosed with liver cirrhosis. This dysbiosis has been observed to detrimentally affect the intestinal barrier’s integrity, resulting in heightened bacterial translocation, infection, systemic inflammation and vasodilation, eventually culminating in acute decompensation of cirrhosis and potential organ failures (Trebicka et al., 2021b). The phenomenon of bacterial translocation originating from the gut microbiota is believed to have a significant role in the development of systemic inflammation and the deterioration of cirrhosis, ultimately impacting the prognosis of individuals with this condition (Simbrunner et al., 2023). The imbalance of gut microbiota and the subsequent translocation of bacteria are significant contributors to the development of chronic liver disorders, including cirrhosis and its associated consequences, such as spontaneous bacterial peritonitis and HE (Arab et al., 2018). As hepatic disorders become more severe, there is a concomitant rise in the frequency and intensity of pathogenic bacterial translocation. Exploring the intricate correlation between gut microbiota and bacterial translocation may offer insights into potential therapeutic targets, which could help alleviate infections and complications related to liver cirrhosis (Wiest et al., 2014). HE is a prominent complication stemming from liver cirrhosis, characterized by changes in the composition and functionality of the gut microbiota. Current therapeutic strategies predominantly focus on modulating the gut microbiota, utilizing treatments such as lactulose, rifaximin, and various other pharmaceutical agents (Hassouneh and Bajaj, 2021). One potential intervention is the administration of lactulose, which has shown beneficial outcomes for patients with minor HE resulting from bacterial-deoxyribonucleic acid (DNA) translocation. Lactulose has the ability to diminish the occurrence of bacterial-DNA translocation and mitigate the subsequent decline in neurocognitive scores (Moratalla et al., 2017). Moreover, the degradation pathways of arginine and ornithine were decreased after lactulose treatment (Wang et al., 2023). In cirrhosis, rifaximin is frequently prescribed to prevent the development of HE (Caraceni et al., 2021). Rifaximin plays a pivotal role in re-establishing the integrity of the intestinal barrier, potentially elucidating its capacity to mitigate bacterial translocation and systemic endotoxemia in patients with liver cirrhosis (Patel et al., 2022). Rifaximin can also decrease the recurrence of HE in individuals with cirrhosis and the risk of hospitalization in HE by regulating the function and activity of the gut microbiota (Bajaj, 2016; Yu et al., 2021a). The study by Shamsaddini et al. (2021) confirmed that rifaximin improves patient outcomes by regulating ARGs in the intestinal microbiome of cirrhotic patients. In conclusion, rifaximin, lactulose, and probiotics can all improve the structure of the gut microbiota to a certain extent and are reliable and efficient in the treatment of minimal HE (Wang et al., 2023). Furthermore, Sharma et al. (2013) found that treatment of HE with both lactulose and rifaximin was superior to lactulose monotherapy.

Keywords grouped within the blue cluster predominantly encompass gut microbiota microbiome dysbiosis bile acids SCFAs hepatocellular carcinoma alcoholic liver disease primary sclerosing cholangitis (PSC) and primary biliary cholangitis (PBC). It has been observed that patients afflicted with diverse liver conditions ranging from alcoholic liver disease non-alcoholic liver disease PSC to PBC consistently exhibit alterations in their gut microbiota. This dysregulation in the intestinal microbial composition can influence the severity of liver steatosis inflammation and fibrosis through various mechanisms (Tilg et al., 2016; Tang et al., 2018). Persistent liver damage can progress to conditions like liver fibrosis eventually resulting in cirrhosis and possibly hepatocellular carcinoma (Kostallari et al., 2021).

Bile acid a crucial metabolite synthesized by the gut microbiota exerts an important impact on the quantity variety and metabolic activities of the intestinal microbiota. The modulation of the bile acid reservoir’s dimensions and composition reciprocally interacts with the configuration of the gut microbiota. The maintenance of this balance is of utmost importance in relation to human health and disease (Ridlon et al., 2013). The impact of bile acid on the absorption or excretion of dietary lipids signifies its potential to modify the composition of the gut microbiome or the metabolic activity of certain bacteria. Consequently regulating the metabolism of bile acid has promise for promoting human health (Collins et al., 2023). Bile acids are instrumental in the pathogenesis of NAFLD (Gottlieb and Canbay, 2019). The primary constituents of the circulating bile acid pool are the secondary bile acids deoxycholic acid and lithocholic acid together with their respective derivatives. The dimensions and constituents of this reservoir play a pivotal role in the management of primary cholangitis and NASH (Funabashi et al., 2020). For example after ursodeoxycholic acid treatment in patients with PBC the abundance of six PBC-related bacterial genera is reversed and gut microbiota emerges as a prospective therapeutic focus and diagnostic biomarker for PBC (Tang et al., 2018). Increasing evidence suggests that the dysregulation of microbial metabolites such as imbalance of secondary bile acids and reduction of SCFA-producing species is central to the pathogenic mechanisms of NAFLD (Albillos et al., 2020; Shashni et al., 2023). Liver cirrhosis patients especially with the decompensation of liver function exhibits serious intestinal microbial dysbiosis. The structure of gut microbiota in persons with this condition differs dramatically from that of healthy people. This is primarily characterized by a recent study that a notable decrease in the abundance of SCFA-producing bacteria such as Firmicutes (which is negatively correlated with liver function indicators) *Coprococcus* and *Clostridium IV* were described (Wu et al., 2023). In addition this study also revealed that the abundance of *Oscillibacter* (also a SCFA-producing bacterium) is markedly reduced accompanied by a notable decrease in the ratio of Firmicutes and Bacteroidetes along with the progression from compensated cirrhosis to decompensated cirrhosis. Further the SCFA-producing Lachnospiraceae family can reduce liver fibrosis and the SCFA-producing families (Ruminococcaceae and Lachnospiraceae) may have a potential association with the progression of HE in cirrhotic patients (Awoniyi et al., 2023). Supplementation with probiotics targeting to enhance the population of SCFA-producing bacteria emerges as a potential therapeutic strategy for cirrhotic patients. In conclusion microbial interventions encompassing adjustments in gut microbiota composition enhancement of SCFA levels and restoration of bile acid metabolism may play a pivotal role in alleviating liver fibrosis (Fu et al., 2022).

The keywords in the green cluster are probiotics, prebiotics, microbiota, endotoxin, antibiotic, cytokines, lactobacillus, TLR, FMT, etc. Probiotics and prebiotics exert their effects via various mechanisms and are instrumental in sustaining the health of the human gut (Sanders et al., 2019; Peng et al., 2020). The therapeutic and prophylactic potential of probiotics and prebiotics in addressing diverse gut-associated ailments may hinge on their ability to alter the composition and functionality of the gut microbiota. The genera *Lactobacillus*, *Bifidobacterium*, and *Saccharomyces*, as probiotics, have been historically documented for their therapeutic efficacy. Meanwhile, *Akkermansia* spp., *Lactobacillus rhamnosus GG*, *Roseburia* spp., *Propionibacterium* spp., and *Faecalibacterium* spp. are promising candidates for future therapeutic applications (Sanders et al., 2019). The treatment of difficulties associated with severe liver illnesses, such as cirrhosis and HE, may be addressed by the administration of a diverse range of prebiotics, probiotics, and antibiotics. This therapeutic approach further underscores the significant contribution of the gut microbiota in the context of liver disorders. Enhanced comprehension of the gut microbiota and its composition in the context of liver disease has the potential to provide a more comprehensive knowledge of these complex conditions, thereby laying the groundwork for new treatment strategies (Tilg et al., 2016).

Previous research has investigated the impact of probiotic strains including several species of *Lactobacillus* and *Bifidobacterium* on the immunological function of the intestinal mucosa and the integrity of the intestinal barrier in individuals diagnosed with NAFLD. Intestinal mucosal immune function may be effectively stabilized by the administration of certain substances, hence offering protection to individuals with NAFLD against heightened permeability resulting from disturbances in the intestinal barrier (Mohamad Nor et al., 2021). Specific strains of *Lactobacillus*, including *L. acidophilus*, *L. fermentum*, and *L. plantarum*, have the potential to mitigate the progression of NASH through cholesterol reduction (Lee et al., 2021). Another randomized clinical trial confirmed that *Lactobacillus GG* can modulate the gut microbiome, metabolome, and endotoxemia in patients with cirrhosis, and the use of *Lactobacillus GG* was safe and well tolerated (Bajaj et al., 2014a). *Lactobacillus lactis* and *Pediococcus pentosaceus* have been shown to ameliorate the progression of NAFLD by regulating both the gut microbiome and metabolome functionalities in mouse models (Yu et al., 2021b). Multiple research investigations have highlighted that *Lactococcus lactis* and *Lactobacillus rhamnosus* have both preventative and therapeutic properties in relation to the advancement of liver fibrosis. Specifically, administration of *Lactobacillus rhamnosus GG* has proven efficacious in counteracting the adverse outcomes of bile acid-induced liver injury and subsequent fibrosis in mouse models. This protective mechanism is achieved via the inhibition of hepatic bile acid production and the promotion of bile acid excretion inside the liver (Liu et al., 2020; Won et al., 2023). In recent advancements, *Akkermansia* spp. is a novel promising probiotic that plays a significant role in immune-mediated liver damage, alcoholic liver disease, and NAFLD (Wu et al., 2017; Grander et al., 2018; Kim et al., 2020).

In recent years, with the development of 16S ribosomal ribonucleic acid (rRNA) sequencing, fecal metagenomic sequencing and other technologies, gut microbiota research has also risen to a new stage. With a more precise and exhaustive analysis of the structural and functional changes of intestinal flora, researchers are capable of revealing the impact of certain species on the occurrence and progression of diseases. In the future, the importance of more gut species may be discovered and become the direction of further research. Besides probiotics, prebiotics, and antibiotics, FMT has also become a potential treatment for diseases related to cirrhosis. It can be used to treat relapsed and refractory *Clostridium difficile* infections, HE, and other conditions. FMT can improve the cognitive ability of patients with perceptual encephalopathy, which is safe and effective (Bajaj et al., 2017; Cheng et al., 2021).

In addition, TLRs can recognize the gut microbiota in cirrhotic patients, triggering signaling cascades linked with the progression of cirrhosis. TLRs hold a crucial position in the occurrence, development, and treatment of cirrhosis (Fan et al., 2021). Furthermore, the gut microbiota and TLR4 are proposed as significant therapeutic targets to prevent hepatocellular carcinoma in individuals with severe liver conditions (Darnaud et al., 2013). TLR4 is also a treatment target for the prevention and management of liver failure (Engelmann et al., 2020). TLRs have multiple functions in the liver, with TLR4 notably fostering oxidative stress, instigating liver inflammation, and driving the progression of fibrosis (Seki et al., 2007; Gill et al., 2010). Numerous natural compounds have demonstrated their potential in attenuating oxidative stress, alleviating liver inflammation, and mitigating fibrosis through the inhibition of the TLR4 signaling pathway or its associated cascades (Luangmonkong et al., 2018).

The primary terms inside the yellow cluster consist of NAFLD, NASH, fatty liver, obesity, metabolic syndrome, oxidative stress, inflammation, fibrosis, insulin resistance, and other related concepts. The gut microbiota is crucial for the overall health and metabolic functions of the host. Dysbiosis, or imbalance within the gut microbiota, has been linked to the onset and progression of numerous metabolic disturbances. These include conditions such as obesity, NAFLD, and insulin resistance (Choi et al., 2021; Fan and Pedersen, 2021). The connection between gut microbiota and host metabolism is modulated by many variables, such as intestinal barrier impairments, bile acid metabolism alterations, antibiotic consumption, and microbial metabolite production. Metabolic syndrome encompasses a cluster of risk factors that, if unmanaged, frequently progress to more severe metabolic complications like type 2 diabetes mellitus and NASH (Dabke et al., 2019). Changes in the composition of gut microbiota can profoundly impact the interaction between intestinal bacteria and the host, mainly by initiating low-grade inflammatory processes leading to the progression of obesity, insulin resistance, NAFLD and other metabolic diseases (Marchesi et al., 2016; Scheithauer et al., 2020). The occurrence of dysbiosis within the gut microbiome, disturbance of the intestinal barrier, and ensuing translocation of bacteria may initiate proinflammatory and profibrotic mechanisms, eventually culminating in the progression of cirrhosis (Pierantonelli and Svegliati-Baroni, 2019; Hyun et al., 2022). Conversely, regulating oxidative stress, fatty acid metabolism, and the balance of gut microbiota may serve as a barrier against hepatic steatosis and fibrosis, subsequently mitigating liver injury (Wang et al., 2021; Nam et al., 2023).

Within the cluster indicated by the color purple, many key components may be identified, including the gut-liver axis, liver fibrosis, intestinal barrier, intestinal microbiota, small intestine bacterial overgrowth, and lipopolysaccharide. The phenomenon of enterohepatic crosstalk has been documented in clinical investigations pertaining to liver disorders, including NAFLD, alcoholic liver disease, and PSC. Cirrhosis often occurs as a consequence of several disorders. Among microbial factors, microbial pathogen-related molecular patterns, for example, are key to driving liver inflammation and clinical complications (Tilg et al., 2022). Both dysbiosis of the intestinal microecology and disturbance of the intestinal barrier have the potential to result in bacterial translocation. Bacterial translocation and leakage of lipopolysaccharide (LPS) have been seen in animal models during the first phases of liver fibrosis. The stimulation of hepatic stellate cells is mediated by LPS via the regulation of autophagy and retinoic acid signaling pathways. Autophagy triggered by LPS can further reduce retinoic acid signaling. This leads to the downregulation of Bambi and promoting the susceptibility of hepatic stellate cells to the fibrotic response of transforming growth factor-β signaling (Chen et al., 2017; Chopyk and Grakoui, 2020). During liver fibrosis, the predominant myofibroblasts present are activated hepatic stellate cells, a distinctive marker of fibrosis (Iwaisako et al., 2014).

As cirrhotic disease progresses, liver transplantation is often eventually required. However, most patients are not eligible for transplantation, and the organ source is scarce. Therefore, further in-depth studies on the regulation of the gut-liver axis are necessary (Bajaj, 2019). In the future, it is necessary to continue to investigate the intricate link between the gut and the liver in clinical trials, which will open up a new way for future targeted therapy.

Specifically, we conduct an independent overlay visualization analysis with keywords of bacterial taxa (Figure 8D). Compared with leading keywords in Figure 8A, most taxa exhibit low occurrence and poor link strength. Studies towards many specific taxa are probably relatively isolated and lacking of extensivity. To be noteworthy, *Lactobacillus*, *Escherichia coli* (*E. coli*) and *Akkermansia muciniphila* (*A. muciniphila*) are three predominant bacterial taxa with both significant occurrence and link strength in this domain. As a contrast, we can observe that *Lactobacillus* and *A. muciniphila* are mainly linked with beneficial species while *E. coli* is mainly linked with pathogens. This is the visual evidence to understand the core species in the both sides (beneficial and harmful) of gut microbiota. In terms of time overlay, we can observe the research trend of these taxa. *Lactobacillus* is a classical probiotic in the research of liver cirrhosis. Many species have exhibited therapeutic potential (Chiva et al., 2002; Liu et al., 2020). *A. muciniphila* is a rising probiotic in recent years and has gained wide acknowledgement in different diseases such as obesity (Depommier et al., 2019), colorectal cancer (Jiang et al., 2023) and liver diseases (Rao et al., 2021). Present findings indicate its therapeutic role in early stage in including NAFLD (Depommier et al., 2019; Rao et al., 2021) and ALD (Grander et al., 2020), whilst its role in advanced liver cirrhosis has not been detected.

Most pathogens, from *Helicobacter pylori* (*H. pylori*), *E. coli* to *Clostridium difficile*, not only indicates the focus of clinical concern, but also reveals the clinical practice from disinfection to microbial regulation. The pathogenic role of *H. pylori* in liver cirrhosis has been widely discussed, which emphasize related disinfection treatment (Wang et al., 2016; Okushin et al., 2018). However, as a common but potentially harmful species, the management of *E. coli* may depend on the balance between disinfection and microbial eubiosis, which provide crucial colonization resistance against *E. coli* (Ducarmon et al., 2019; Zhang et al., 2022). Further, *Clostridium difficile* infection has raised clinical concern of excessive antibiotic use, which can unfavorably induce dysbiosis, leading the outburst of the resistant bacteria (Martin et al., 2016). The academic trend here represents the advancement of the clinical understanding and practice: in some scenarios, compared with simple eradication of harmful species, the promotion of beneficial community is as well critical.

## 5 Summary

This study has the following advantages: our study is the first to systematically and comprehensively analyze the gut microbiota of liver cirrhosis using bibliometric methods, which is different from previous studies. For example, Pezzino S et al. undertook a bibliometric study on the impact of the intestinal microbiome in NAFLD/NASH, and related liver cirrhosi based on the Dimensions scientific research database (Pezzino et al., 2023). Our study includes research on cirrhosis also caused by viral hepatitis, autoimmune diseases, long-term heavy alcohol consumption, obesity, cholestasis, and other causes. Our study is helpful to fully reveal the role of intestinal microbiology in this multi-etiological pathological process of liver cirrhosis because the patterns of gut microbiota in liver cirrhosis with different causes are not completely the same.

This investigation is not without its limitations. Firstly, the exclusive reliance on the WoSCC database might have inadvertently omitted relevant research from alternative databases. Furthermore, the study’s focus on solely English publications could have led to the exclusion of significant research in other languages. This omission could potentially affect the comprehensiveness of our findings.

In summary, this work represents the first thorough bibliometric analysis conducted on publications pertaining to the investigation of gut microbiota in patients diagnosed with cirrhosis resulting from various etiologies. Recent years have witnessed a burgeoning interest in the gut microbiota’s relationship with liver cirrhosis, mirroring the advancements in our understanding of the pathophysiological processes of liver cirrhosis. The gut microbiota is known to have a substantial effect on the development and advancement of liver cirrhosis, along with its associated consequences. The efficacy of therapeutic interventions aimed at modulating the gut microbiota has been demonstrated in patients diagnosed with liver cirrhosis, regardless of accompanying comorbidities. Hence, a comprehension of the intestinal flora’s mechanism in liver cirrhosis of diverse etiologies establishes the foundation and offers substantiation for the prospective treatment of liver cirrhosis through the selective targeting of specific bacteria. This approach has the potential to enhance patient prognosis and alleviate the societal burden associated with chronic liver disease. Furthermore, it is essential for organizations and nations to actively participate in international cross-border collaboration in order to facilitate the progress of gut microbiota in the context of liver cirrhosis.

## Data availability statement

The original contributions presented in the study are included in the article/supplementary material, further inquiries can be directed to the corresponding author.

## Author contributions

XZ: Writing – original draft. ZZ: Writing – original draft. XP: Writing – review & editing.
